# TRRAP is a central regulator of human multiciliated cell formation

**DOI:** 10.1083/jcb.201706106

**Published:** 2018-06-04

**Authors:** Zhao Wang, Lindsey W. Plasschaert, Shivani Aryal, Nicole A. Renaud, Zinger Yang, Rayman Choo-Wing, Angelica D. Pessotti, Nathaniel D. Kirkpatrick, Nadire R. Cochran, Walter Carbone, Rob Maher, Alicia Lindeman, Carsten Russ, John Reece-Hoyes, Gregory McAllister, Gregory R. Hoffman, Guglielmo Roma, Aron B. Jaffe

**Affiliations:** 1 Chemical Biology and Therapeutics, Novartis Institutes for BioMedical Research, Cambridge, MA; 2 Analytical Sciences and Imaging, Novartis Institutes for BioMedical Research, Cambridge, MA; 3 Novartis Institutes for BioMedical Research, Novartis Pharma AG, Basel, Switzerland

## Abstract

The multiciliated cell (MCC) is an evolutionarily conserved cell type, which in vertebrates functions to promote directional fluid flow across epithelial tissues. In the conducting airway, MCCs are generated by basal stem/progenitor cells and act in concert with secretory cells to perform mucociliary clearance to expel pathogens from the lung. Studies in multiple systems, including *Xenopus laevis* epidermis, murine trachea, and zebrafish kidney, have uncovered a transcriptional network that regulates multiple steps of multiciliogenesis, ultimately leading to an MCC with hundreds of motile cilia extended from their apical surface, which beat in a coordinated fashion. Here, we used a pool-based short hairpin RNA screening approach and identified TRRAP, an essential component of multiple histone acetyltransferase complexes, as a central regulator of MCC formation. Using a combination of immunofluorescence, signaling pathway modulation, and genomic approaches, we show that (a) TRRAP acts downstream of the Notch2-mediated basal progenitor cell fate decision and upstream of Multicilin to control MCC differentiation; and (b) TRRAP binds to the promoters and regulates the expression of a network of genes involved in MCC differentiation and function, including several genes associated with human ciliopathies.

## Abstract (precis)

Multiciliated cells (MCCs) function to promote directional fluid flow across epithelial tissues. Wang et al. show that TRRAP, a component of multiple histone acetyltransferase complexes, is required for airway MCC formation and regulates a network of genes involved in MCC differentiation and function.

## Introduction

A key function of epithelial tissues is to act as protective barriers between the body and the environment. This is exemplified by the respiratory tract, which is bombarded by airborne pathogens and particulates with every breath. In the airway, the two major differentiated epithelial cell types, secretory and ciliated cells, act together to perform mucociliary clearance, trapping and expelling pathogens from the airway (Bustamante-Marin and Ostrowski, 2017). Secretory and ciliated cells are generated from a common progenitor, the airway basal cell (Rock et al., 2009). The lineage decision between secretory and ciliated cells is tightly regulated during development, homeostasis, and regeneration (Hogan et al., 2014). An imbalance in the abundance of these two differentiated cell types, leading to goblet cell metaplasia and increased mucus production, is seen in a variety of airway diseases, such as asthma, chronic obstructive pulmonary disease, and cystic fibrosis (Fahy and Dickey, 2010).

Notch signaling has emerged as a key pathway controlling the secretory versus ciliated lineage decision. Notch signaling is an evolutionarily conserved pathway that regulates many lineage fate decisions (Fortini, 2009). In the developing airway, Notch activation is sufficient to drive secretory cell formation at the expense of ciliated cells (Guseh et al., 2009), whereas inhibition of Notch signaling leads to an increase in the number of ciliated cells and a concomitant decrease in secretory cell formation (Tsao et al., 2009). Notch2 is crucial for lineage decisions in the airway, as deletion of *Notch2*, but not *Notch1* or *Notch3*, in the developing lung leads to increased ciliated cell abundance and decreased secretory cell abundance (Morimoto et al., 2012), and inhibition of Notch2 with receptor-specific antibodies prevents goblet cell metaplasia driven by diverse stimuli in primary human airway cell cultures in vitro and mouse airways in vivo (Danahay et al., 2015; Lafkas et al., 2015).

In addition to the Notch pathway, epigenetic regulation also influences stem and progenitor cell fate decisions (Lunyak and Rosenfeld, 2008; MuhChyi et al., 2013; Roostaee et al., 2016). In the developing mouse lung, deletion of *Ezh2*, a Polycomb group protein that serves as the catalytic subunit of Polycomb repressive 2 complex for H3K27 methylation, leads to ectopic and premature formation of basal cells and a reduction of secretory cells (Snitow et al., 2015), whereas deletion of histone deacetylases 1 and 2 inhibits proximal airway development (Wang et al., 2013). Whether additional epigenetic factors regulate airway progenitor lineage decisions is unknown.

Once the lineage choice is made by the airway basal cell, it must fully mature into a differentiated, specialized cell type that performs distinct functions. For example, after committing to a multiciliated cell (MCC) fate, a transcriptional hierarchy drives a series of intracellular events including centriole multiplication, formation of basal bodies, docking of basal bodies at the apical surface, and ultimately the extension of hundreds of cilia, which beat in a coordinated fashion (Brooks and Wallingford, 2014). Whether and how epigenetic factors regulate differentiation steps downstream of lineage decisions is unclear.

To uncover epigenetic mechanisms that regulate airway epithelial cell fate decisions, we established a new method to perform a pooled shRNA screen against known epigenetic regulators. The screen uncovered TRRAP as a specific and essential component of efficient MCC formation from human airway basal cells.

## Results and discussion

### A pooled shRNA screen uncovers epigenetic regulators of airway epithelial cell fate

**Figure 1.**
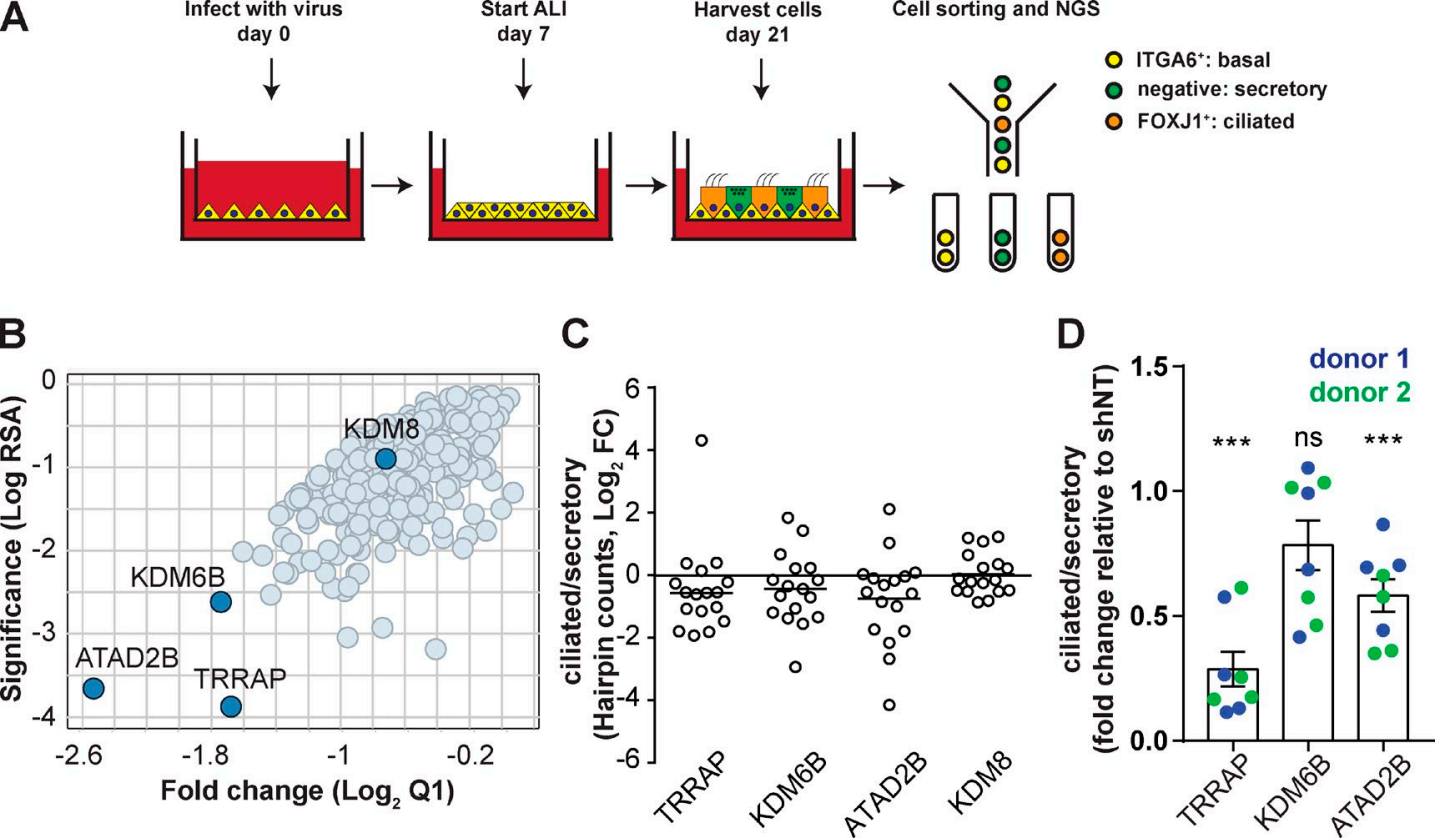
**A pooled shRNA screen to identify regulators of basal cell fate. (A)** Workflow of the pooled shRNA screen. Human airway basal cells were transduced with a lentiviral shRNA library 1 h after they were seeded on Transwell filters at day 0. At day 21, cells were fixed, stained, and sorted into three different groups based on expression of the ciliated cell marker FOXJ1 and the basal cell marker ITGA6. The extracted gDNA from the sorted samples was analyzed for shRNA hairpin counts by NGS. **(B)** Gene-centric visualization of the log2 fold change and RSA score in the ciliated (ITGA6^−^ FOXJ1^+^) versus secretory (ITGA6^−^ FOXJ1^−^) cells. **(C)** Ratio of individual shRNA hairpin counts in ciliated/secretory cells from the pooled shRNA screen. KDM8 is shown as an example of a gene whose shRNA barcode counts do not differ between ciliated and secretory cells. **(D)** In a primary validation, the cells from two independent donors were transduced with four shRNAs for each gene candidate and sorted with FOXJ1 and ITGA6 signals. The ratios of ciliated/secretory cells from all shRNA-treated samples were normalized to those of control cells transduced with shNT. Data were acquired from cells of two donors; mean ± SEM; ***, P < 0.001; Student’s two-tailed *t* test.

To identify genes required for airway epithelial cell fate decisions, we established a pooled shRNA screening strategy in primary human airway basal cells, the progenitor cells in the conducting airway. The strategy used a lentiviral shRNA library of 4792 shRNAs targeting 281 genes encoding proteins involved in epigenetic regulation, or with domains known for this function (Hoffman et al., 2014; Table S1). A schematic of the method is in Fig. 1 A. Human airway basal cells were infected at the beginning of cell culture (day 0), differentiated at air–liquid interface (ALI), and sorted into three different populations on day 21. To isolate differentiated cell types, we adapted a recently described sorting strategy using both intracellular and cell surface markers (Hrvatin et al., 2014). Cells were stained for FOXJ1, a transcription factor expressed in ciliated cells, and ITGA6, an integrin subunit enriched in basal cells, and separated into three populations by FACS: basal cells (ITGA6^+^ FOXJ1^−^, referred to as ITGA6^+^ hereafter), ciliated cells (ITGA6^−^ FOXJ1^+^, referred to as FOXJ1^+^ hereafter), and secretory cells (ITGA6^−^ FOXJ1^−^). We validated our sorting strategy with fully differentiated, nontransduced ALI cultures by quantitative RT-PCR (qPCR) for cell type–specific markers (Fig. S1, A and B). After sorting our screening samples, we harvested genomic DNA (gDNA) for next-generation sequencing (NGS) to count shRNA barcodes in each cell type (Fig. 1 A). In this method, shRNAs targeting genes required for a lineage fate decision would become underrepresented in the barcode counts from a specific cell population. Comparing barcode counts in the differentiated populations (ciliated and secretory cells) would identify genes required for differentiation into either cell type. Hairpins targeting three genes, *TRRAP*, *ATAD2B*, and *KDM6B*, were markedly depleted in ciliated cells, suggesting that they function in MCC formation (Fig. 1, B and C).

To validate these three hits, we silenced each with four individual shRNAs in airway basal cells from two independent human donors. The cells were differentiated at ALI, stained for cell type–specific markers as above, and analyzed by flow cytometry. Silencing either *TRRAP* or *ATAD2B* reduced the ratio of ciliated to secretory cells, whereas silencing *KDM6B* did not result in a significant change. These data confirmed the two strongest hits from the primary screen and suggested a role for *TRRAP* and *ATAD2B* in ciliated cell formation (Fig. 1 D). *TRRAP* was pursued in further studies because its silencing had the greater impact on the ratio of ciliated/secretory cells.

### TRRAP is required for ciliated cell formation, but not secretory cell formation

**Figure 2.**
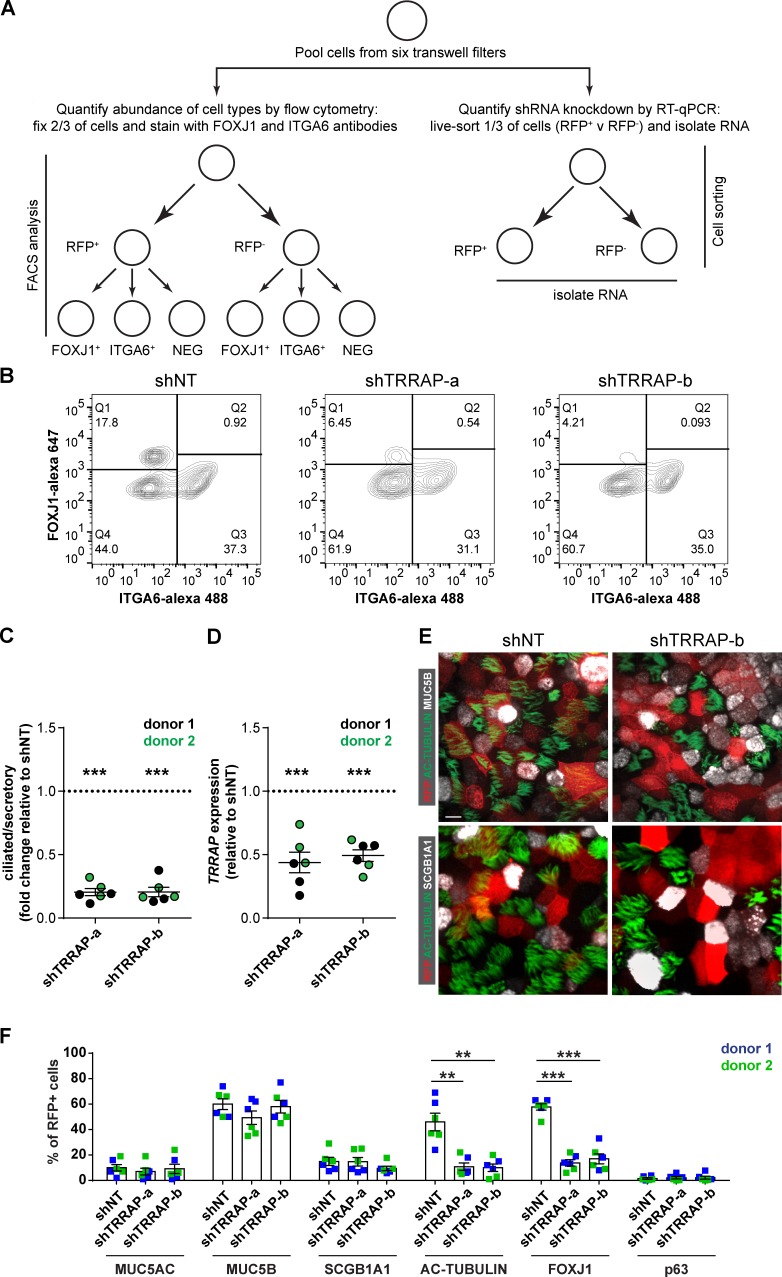
**TRRAP is required for MCC formation. (A)** Schematic workflow for validation of TRRAP. Six Transwell filters of fully differentiated ALI cultures derived from cells transduced with either shNT or *TRRAP* shRNA (shTRRAP) were pooled together. Two thirds were fixed and stained for ciliated (FOXJ1) and basal (ITGA6) markers and analyzed by flow cytometry. The remaining third was sorted with RFP to estimate gene knockdown by qPCR. **(B)** Representative FACS plots from *TRRAP* shRNA treatment. Human airway basal cells transduced with viruses for either shNT or *TRRAP* shRNAs were fixed and sorted with FOXJ1 and ITGA6 signals. **(C)** Ratios of ciliated/secretory cells in the *TRRAP* shRNA–treated samples were normalized to the ones from NT controls. **(D)** Quantification of *TRRAP* knockdown. CT values from qPCR in the *TRRAP* shRNA transduced cells were normalized to those from shNT controls (*n* = 6 from two independent donors; mean ± SEM; ***, P < 0.001). **(E)** The cells transduced with either shNT or shTRRAP were immunostained for markers of goblet cells (MUC5AC, MUC5B), club cells (SCGB1A1), ciliated cells (AC-TUBULIN, FOXJ1), or basal cells (p63). Representative images from ALI cultures immunostained for AC-TUBULIN/MUC5B (top) or AC-TUBULIN/SCGB1A1 (bottom) are shown. Bar, 10 µm. **(F)** The RFP^+^ cells expressing the indicated cell markers were normalized to the total RFP^+^ cells to calculate the percentage of goblet, club, ciliated, or basal cells after *TRRAP* knockdown or in shNT controls. *n* = 6 from two independent donors; mean ± SEM; **, P < 0.01; ***, P < 0.001; Student’s two-tailed *t* test.

*TRRAP* is a common subunit of multiple transcriptional coactivator complexes (Murr et al., 2007) and is essential for MYC-driven transformation (McMahon et al., 1998). However, a role for TRRAP in MCC formation has not been described. To further validate this new role for *TRRAP*, we first determined whether the effect of *TRRAP* silencing correlated with loss of the *TRRAP* transcript (Fig. 2 A). We infected cells with lentiviruses encoding the two shRNAs that had the strongest effect on the ratio of ciliated to secretory cells (Fig. 1 D) and allowed the cells to differentiate at ALI. We then harvested the cells and analyzed one-third of them by qPCR, confirming a reduction in *TRRAP* mRNA expression in cells infected with *TRRAP* shRNAs compared with cells infected with a nontargeting shRNA control (shNT; Fig. 2 D). We fixed and stained the remaining two-thirds of the cells for markers of basal and ciliated cells and analyzed the relative abundance of secretory and ciliated cells by flow cytometry. The flow cytometric analysis revealed a significant reduction in the ratio of ciliated to secretory cells by each of the *TRRAP* shRNA treatments (Fig. 2, B and C), consistent with the primary screening results. The altered ratio was driven by a decrease in the percentage of ciliated cells (FOXJ1^+^, ITGA6^−^), with a concomitant increase in the percentage of secretory cells (FOXJ1^−^ and ITGA6^−^), without markedly affecting the percentage of basal cells (FOXJ1^−^ and ITGA6^+^). Because our sorting method classifies secretory cells based on the absence of ciliated and basal cell markers, these results are consistent with at least two hypotheses: (1) TRRAP knockdown biases airway basal cell fate away from ciliated cells and toward the secretory cell lineage, or (2) TRRAP knockdown results in a failure to fully differentiate into MCCs, without affecting progenitor cell lineage commitment.

To distinguish between these possibilities, we transduced cells with shNT or shRNAs targeting TRRAP, differentiated the cells at ALI, and immunostained for markers of two secretory cell populations, goblet cells (MUC5B or MUC5AC) and club cells (SCGB1A1), as well as ciliated cells (acetylated α-tubulin [AC-tubulin] or FOXJ1). We then quantified the fraction of shRNA-expressing cells, as indicated by RFP expression, that were secretory or ciliated (Fig. 2, E and F; and Fig. S1, C and D). Knockdown of TRRAP decreased the number of ciliated cells as judged by AC-tubulin or FOXJ1 staining. Interestingly, we saw no effect on SCGB1A1-, MUC5AC-, or MUC5B-positive secretory cell numbers, indicating that *TRRAP* is dispensable for secretory cell formation.

### TRRAP is expressed before FOXJ1 in ciliated cells

**Figure 3.**
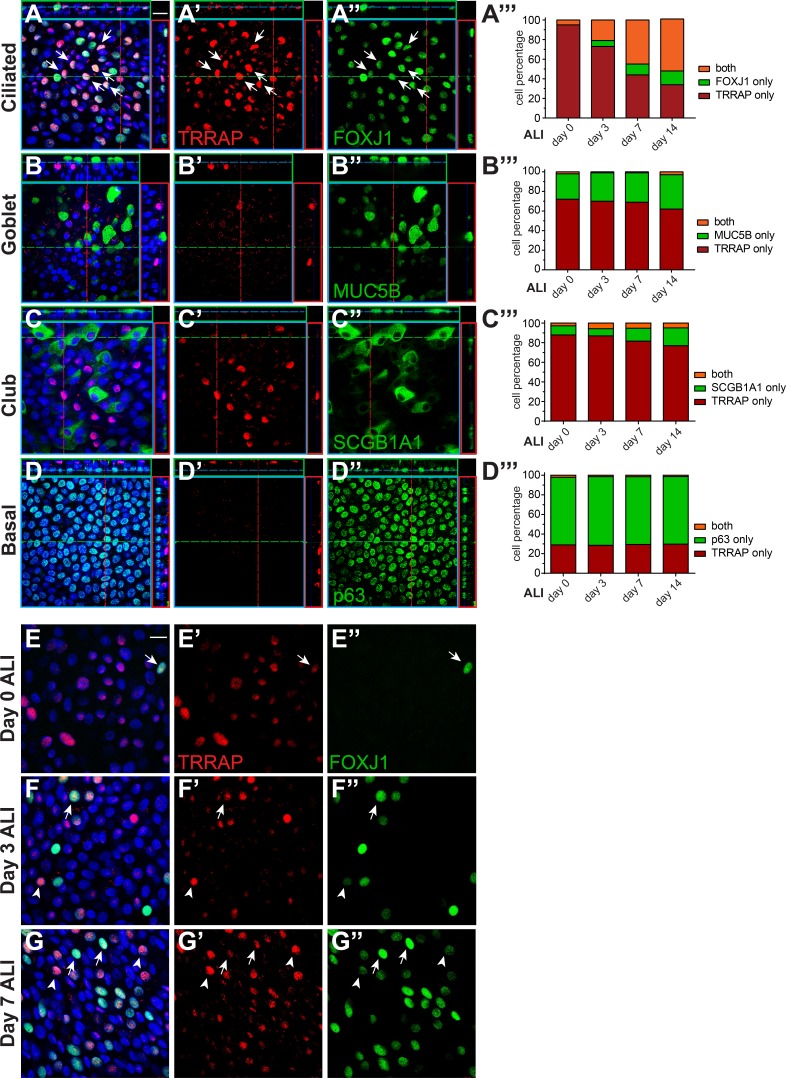
**TRRAP is enriched in ciliated cells and accumulates before FOXJ1. (A–D)** During the course of differentiation at ALI, cultures were stained with antibodies to TRRAP, FOXJ1 (ciliated cells), MUC5B (goblet cells), SCGB1A1 (club cells), and p63 (basal cells). Colocalization was quantified at days 0, 3, 7, and 14 at ALI (A′′′–D′′′). At ALI day 14, TRRAP colocalizes with FOXJ1 in ciliated cells (arrows in A–A′′) but rarely accumulates in other cell types (A′–D′′). **(E–G)** At day 0, before culture at ALI, TRRAP has begun to accumulate in nuclei with a small subset of those being coenriched for FOXJ1 (arrows; A′′′; arrow in E–E′′). At days 3 and 7 of culture at ALI, the number of cells enriched for both TRRAP and FOXJ1 increases (A′′′; arrows in F–G′′), and the number of TRRAP-only cells decreases. Notably, cells with high TRRAP accumulation have low FOXJ1 (F–G′′, arrowheads), whereas cells with high FOXJ1 accumulation have low TRRAP (F–G′′, arrows), suggesting that their expression is temporally distributed in the same cell type. Bars, 20 µm.

To better define the cell type and stage in which TRRAP acts in airway epithelial cell differentiation, we examined its temporal and spatial expression pattern during ALI differentiation in vitro. We performed coimmunofluorescence studies with antibodies to TRRAP and the cell type–specific markers for ciliated cells (FOXJ1), goblet cells (MUC5B), club cells (SCGB1A1), and basal cells (p63) at 0, 3, 7, and 14 d at ALI (Fig. 3). We verified the specificity of the TRRAP antibody by examining the staining pattern in cells transduced with shTRRAP or shNT (Fig. S2).

1 wk after plating the cells on Transwell filters (day 0 ALI), a subset of suprabasal cells showed enriched TRRAP expression, with 5% of these TRRAP-enriched cells expressing FOXJ1 (Fig. 3, A′′′ and E–E′′). As cells differentiated, this double-positive population (TRRAP^+^ FOXJ1^+^) increased concomitant with a decrease in the fraction of cells expressing TRRAP alone (TRRAP^+^ FOXJ1^−^) and emergence of a population enriched for FOXJ1 alone (TRRAP^−^ FOXJ1^+^) visible at days 3 and 7 at ALI (Fig. 3, A′′′, F, and G′′). By day 14 at ALI, when the culture had fully differentiated, the double-positive population increased to 53%, whereas the population of cells that expressed TRRAP alone decreased to 34% (Fig. 3, A–A′′′). In addition to TRRAP accumulating before FOXJ1, we noted that TRRAP appears highest in cells with low FOXJ1, and FOXJ1 appears highest in cells with low TRRAP, demonstrating a temporal relationship and suggesting that TRRAP acts early in MCC differentiation. In contrast, we observed very few cells coexpressing TRRAP and the secretory cell markers MUC5B or SCGB1A1 at any time point (Fig. 3, B–C′′′). Although we observed low levels of TRRAP staining in all p63^+^ basal cells shortly after seeding (not depicted), we did not detect TRRAP in p63^+^ basal cells at any time during differentiation at ALI (Fig. 3, D–D′′′). These data suggest that TRRAP acts in MCC formation before FOXJ1.

### TRRAP acts downstream of Notch 2 signaling to regulate MCC formation

**Figure 4.**
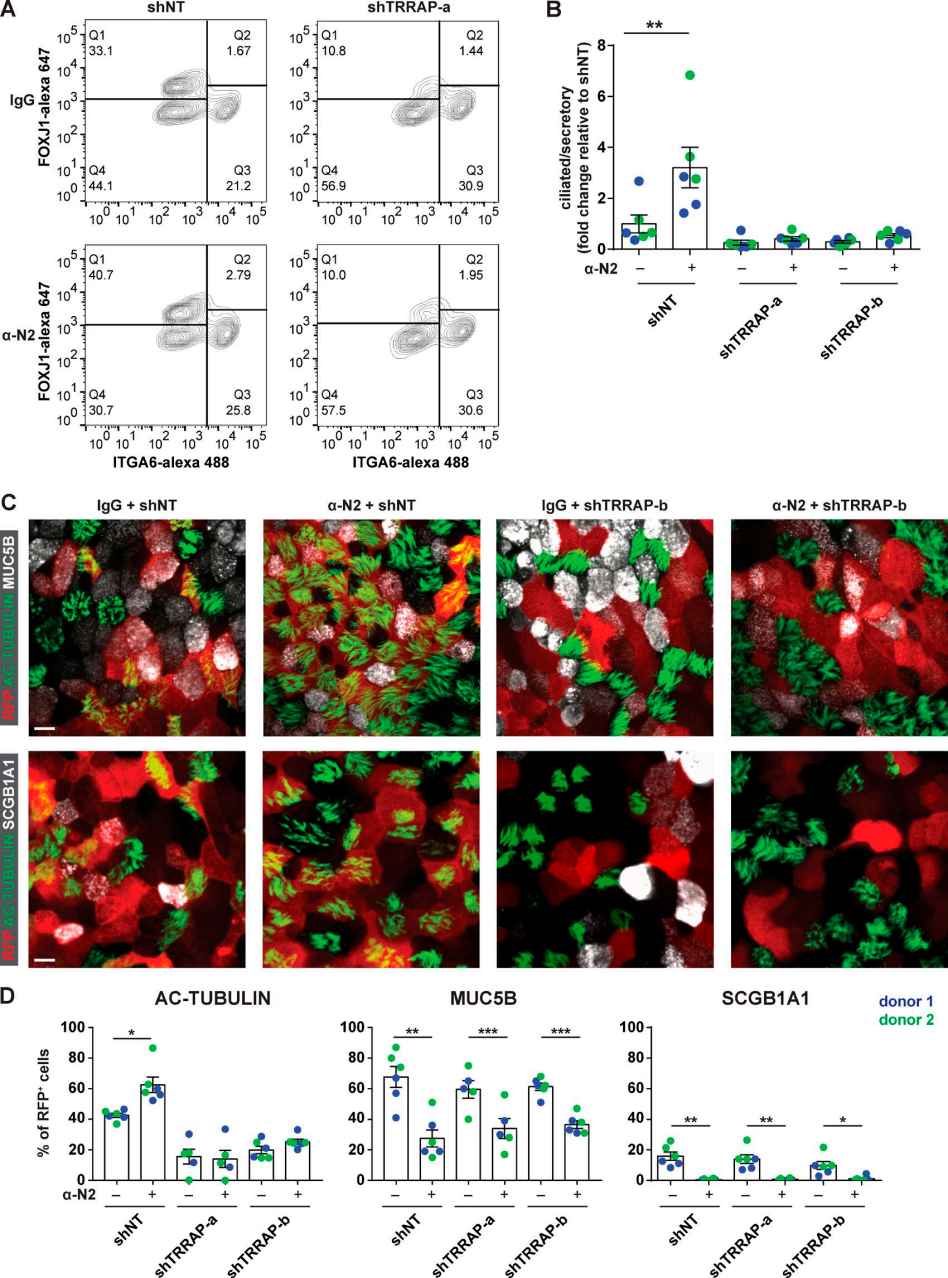
**TRRAP acts downstream of Notch2 signaling to regulate MCC formation. (A)** Representative FACS plots of shRNA-infected ALI cultures that were treated with IgG or α-N2. Human airway basal cells were transduced with either shNT or shTRRAP-a at day 0. The cells were cultured in medium containing either 1 µg/ml IgG or α-N2 from day 7 (ALI day 0). At day 21, cells were stained for FOXJ1 and ITGA6 to label ciliated and basal cells, respectively, and analyzed by flow cytometry. **(B)** The ratio of ciliated/secretory cells of each sample was normalized to the control cells treated with IgG and shNT (*n* = 6 from two independent donors; mean ± SEM). **(C)** Representative images of ALI cultures with the indicated treatments, stained for AC-TUBULIN, to label ciliated cells, and either MUC5B (top row) or SCGB1A1 (bottom row) to label goblet or club cells, respectively. Bar, 10 µm. **(D)** Quantification of the percentage of RFP^+^ (shRNA-containing) ciliated (AC-TUBULIN^+^), goblet (MUC5B^+^), and club (SCGB1A1^+^) cells. Data were obtained from six independent cultures with two independent donors. Error bars indicate SEM. *, P < 0.05; **, P < 0.01; ***, P < 0.001; Student’s two-tailed *t* test.

Notch 2 signaling is critical for the lineage decision between secretory and ciliated cell fates in the conducting airway, as inhibition of Notch 2 by genetic deletion or antibody treatment leads to increased numbers of ciliated cells at the expense of secretory cells (Morimoto et al., 2012; Danahay et al., 2015; Lafkas et al., 2015). We therefore tested the epistatic relationship between TRRAP and Notch 2 by cotreating cells with a Notch 2 inhibitory antibody and TRRAP shRNAs. By flow cytometric analysis, we found that in the presence of control IgG, the two TRRAP shRNAs reduced the ratio of ciliated to secretory cells (Fig. 4, A and B). In cells infected with shNT, Notch 2 antibody treatment increased the ratio of ciliated to secretory cells by 3.2-fold compared with cells treated with IgG (Fig. 4, A and B). However, the effect of Notch 2 inhibition was abolished by cotreatment with TRRAP shRNA, phenocopying treatment with IgG and shTRRAP (Fig. 4, A and B). This finding indicates that TRRAP acts downstream of Notch 2 to regulate MCC formation.

We then performed immunostaining of ALI cultures with antibodies to AC-tubulin, MUC5B, and SCGB1A1 to label ciliated cells, goblet cells, and club cells, respectively (Fig. 4, C and D; and Fig. S3, A and B). In control IgG-treated cultures, TRRAP shRNAs reduced the abundance of ciliated cells without affecting goblet or club cell abundance, whereas anti-N2 treatment increased ciliated cell numbers and decreased secretory cell numbers in cultures expressing shNT (Fig. 4 D). Cotreatment with anti-N2 antibodies and each of the TRRAP shRNAs reduced secretory cell numbers but failed to increase ciliated cell numbers, phenocopying shTRRAP treatment alone (Fig. 4 D). This finding is consistent with our flow cytometry results and further supports the model that TRRAP acts downstream of N2 to drive MCC formation, without affecting the airway basal cell lineage choice between a secretory and ciliated cell. Consistent with this model, TRRAP silencing did not alter the fraction of MUC5B^+^ goblet cells or SCGB1A1^+^ club cells in cultures treated with anti-Notch2 antibodies.

### TRRAP acts upstream of Multicilin in controlling MCC formation

**Figure 5.**
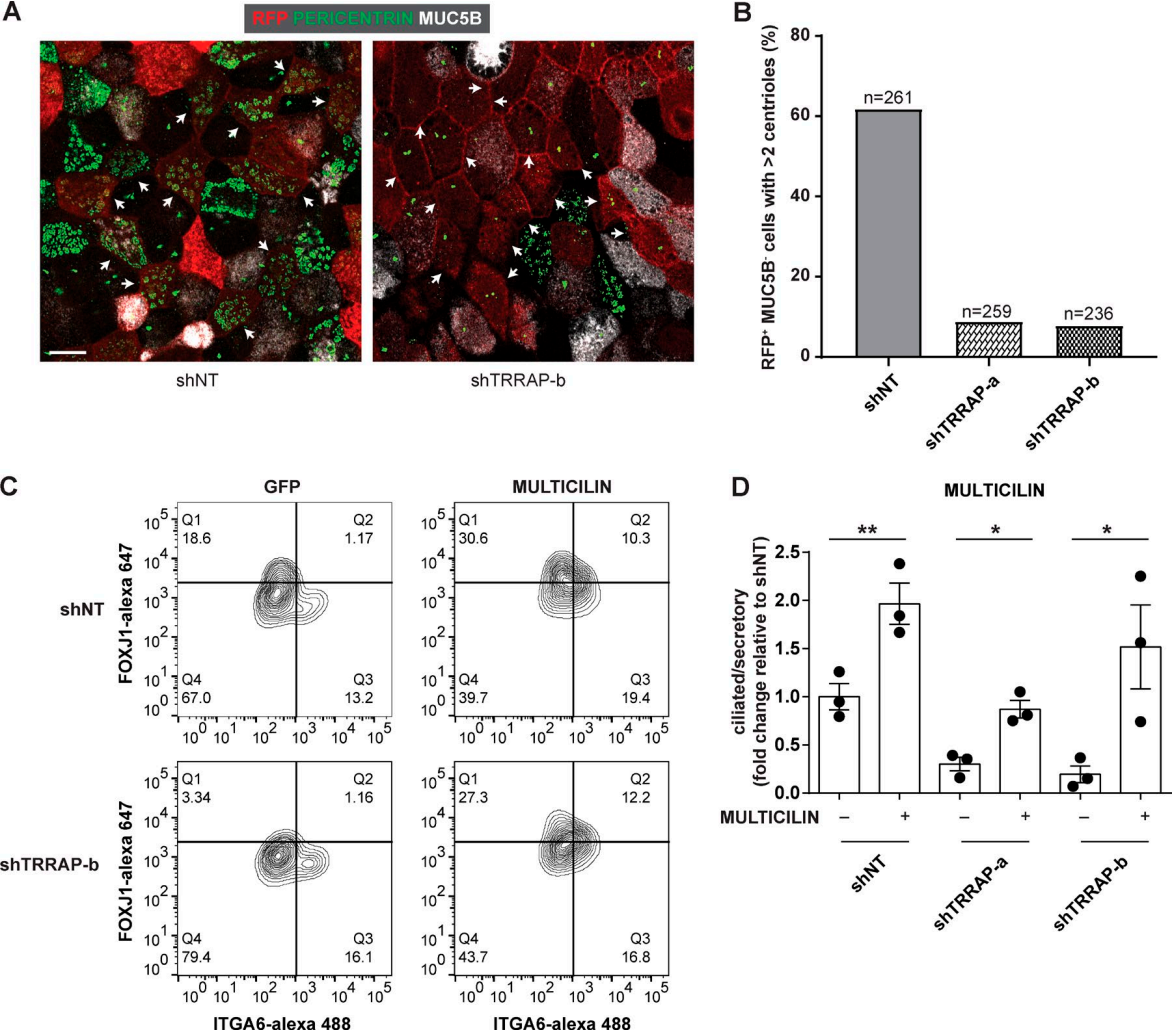
**TRRAP regulates MCC formation upstream of MULTICILIN. (A)** Human airway basal cells transduced with either shNT or shTRRAP-b at day 0 were differentiated at ALI and stained for RFP (red), PERICENTRIN (green), or MUC5B (white). Arrowheads highlight RFP^+^MUC5B^−^ presumptive ciliated cells. Note that knockdown of TRRAP results in an inhibition of centriole replication. Bar, 10 µm. **(B)** Quantification of the percentage of shRNA-expressing presumptive ciliated cells (RFP^+^ MUC5B^−^) with more than two centrioles. Each condition was performed in two independent donors, and the total number of cells quantified is indicated. **(C)** Representative FACS plots of day 21 ALI cultures, which had been transduced with lentivirus encoding for *GFP* or *MULTICILIN-GFP*, with either shNT or shTRRAP-b at day 0. Cultures were dissociated and then sorted based on FOXJ1 and ITGA6 signals. **(D)** Quantification of the ratio of ciliated/secretory cells from the indicated treatments, normalized to control cells that were cotransduced with shNT and *GFP* (*n* = 3). Data shown are represented as mean ± SEM; *, P < 0.05; **, P < 0.01; Student’s two-tailed *t* test.

Multicilin is necessary and sufficient for MCC formation in *Xenopus laevis* and mouse (Stubbs et al., 2012; Ma et al., 2014), and mutations in *MCIDAS*, the gene encoding for Multicilin, cause the mucociliary clearance disorder called reduced generation of multiple motile cilia (Boon et al., 2014). Multicilin regulates centriole assembly (Stubbs et al., 2012), a very early step in MCC differentiation (Brooks and Wallingford, 2014). To examine the relationship between TRRAP and Multicilin, we first examined the effect of TRRAP knockdown on centriole assembly in differentiating MCCs. Because our previous data indicated that TRRAP is required for FOXJ1 expression, we analyzed presumptive MCCs by staining fully differentiated ALI cultures with MUC5B to mark secretory cells and analyzed centriole assembly in MUC5B^−^ RFP^+^ cells at the apical surface of the culture (Fig. 5 A). Silencing TRRAP reduced the number of presumptive MCCs containing multiple apically localized centrioles (Fig. 5 B), phenocopying the previously reported effect of Multicilin inhibition (Stubbs et al., 2012).

Next, we performed epistasis experiments to determine whether TRRAP acts upstream or downstream of Multicilin. We first examined whether Multicilin was sufficient to induce ciliated cell formation by overexpressing either GFP or GFP-Multicilin in airway basal cells and differentiating them at ALI. Compared with GFP control, overexpression of Multicilin increased the ratio of ciliated/secretory cells in fully differentiated ALI cultures (day 21; Fig. 5, C and D), indicating that Multicilin is sufficient to drive MCC formation in our system. We next tested whether overexpression of Multicilin can rescue the loss of ciliated cells by *TRRAP* knockdown. In the cells expressing GFP, *TRRAP* silencing reduced the ratio of ciliated/secretory cells compared with the cells infected with shNT. Overexpression of GFP-Multicilin in the background of *TRRAP* knockdown restored the ratio of ciliated/secretory cells to levels similar to or higher than those seen in control cells expressing GFP and shNT (Fig. 5, C and D). These data indicate that TRRAP acts upstream of Multicilin in regulating ciliated cell formation.

### TRRAP directly regulates the expression of genes required for MCC development and function

**Figure 6.**
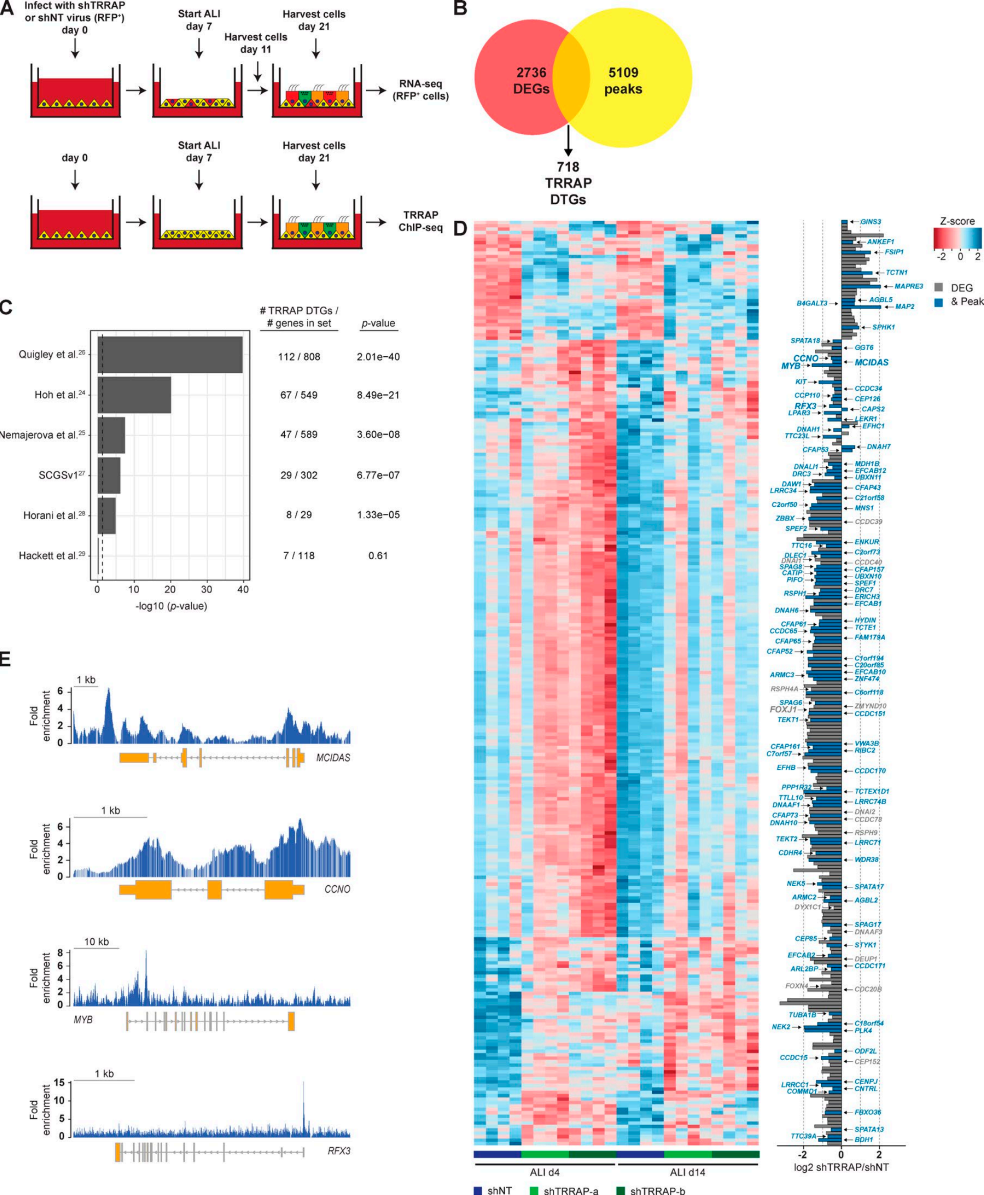
**TRRAP regulates a network of genes controlling multiple steps of multiciliogenesis. (A)** Schematic of the experimental design. **(B)** Venn diagram of the number of TRRAP DEGs, identified by RNA-seq, and the number of promoter regions (<1 kb from the transcription start site) containing TRRAP peaks. **(C)** Enrichment analysis of TRRAP DTGs in the indicated datasets. The number of DTGs within each dataset is indicated. Shown are the p-values calculated by Fisher’s exact test with Bonferroni correction. Note the TRRAP DTG enrichment in sets of MCC genes (Hoh et al., 2012; van Dam et al., 2013; Nemajerova et al., 2016; Quigley and Kintner, 2017) and genes that are mutated in PCD; (Horani and Ferkol, 2016) but not in the set of basal cell genes (Hackett et al., 2011). **(D)** Hierarchical clustering of the 271 ciliogenesis DEGs at ALI days 4 and 14 in shNT, shTRRAP-a, and shTRRAP-b samples. Shown are the *z*-scores for each gene across all samples. Right: Bar plot displaying the maximum log2 expression differences between shTRRAP and shNT samples. Gray, DEGs; blue, DEGs containing a TRRAP peak in the promoter. **(E)** ChIP-seq tracks displaying TRRAP binding at the *MCIDAS*, *CCNO*, *MYB*, and *RFX3* genomic loci. Shown is the fold enrichment over input.

Because TRRAP is an essential component of multiple transcriptional coactivator complexes, we reasoned that the phenotypic consequence of knocking down TRRAP was caused by altered gene expression. To explore this possibility, we performed RNA sequencing (RNA-seq) and chromatin immunoprecipitation sequencing (ChIP-seq) on fully differentiated ALI cultures (Fig. 6 A). We identified 2,736 differentially expressed genes (DEGs) in shTRRAP cells compared with cells expressing shNT. 718 of these had TRRAP bound in the promoter region (Fig. 6 B and Table S3), suggesting they are directly regulated by TRRAP-containing complexes (direct target genes [DTGs]). We next compared the 718 TRRAP DTGs with previously published lists of MCC genes (Hoh et al., 2012; van Dam et al., 2013; Nemajerova et al., 2016; Quigley and Kintner, 2017), genes that are mutated in primary ciliary dyskinesia (PCD; Horani and Ferkol, 2016), and a list of basal cell genes (Hackett et al., 2011) and found a significant enrichment of TRRAP DTGs in the MCC and PCD gene sets, but not in the basal cell gene set (Fig. 6 C). To determine how TRRAP regulates MCC formation, we compared our DEG gene set with a list of “core MCC genes” (Quigley and Kintner, 2017). Of the 808 annotated core MCC genes, 271 were DEGs and 112 were DTGs (Fig. 6 D). Interestingly, three of the TRRAP DTGs, *MCIDAS*, *CCNO*, and *MYB* (Fig. 6, D and E; and Fig. S3 C), have been previously shown to be required for centriole replication (Stubbs et al., 2012; Tan et al., 2013; Wallmeier et al., 2014; Funk et al., 2015), and *MCIDAS*, *MYB*, and another TRRAP DTG, *RFX3*, have been reported to regulate *FOXJ1* expression (El Zein et al., 2009; Stubbs et al., 2012; Tan et al., 2013). Together, these data provide a molecular mechanism for our observed phenotypic effects of TRRAP knockdown on MCC formation. Interestingly, of the 29 PCD-associated genes (Horani and Ferkol, 2016), 19 are TRRAP DEGs and 8 also have a TRRAP peak in the promoter region (Fig. 6 D). In addition, we identified several other classes of MCC genes, including those regulating aspects of cilia structure and function, as TRRAP DTGs and DEGs (Fig. 6 D). Collectively, our results suggest that TRRAP regulates a broad network of genes required for MCC differentiation and function.

Here, we demonstrate that *TRRAP* is required for MCC formation in human airway epithelial cells. We propose that TRRAP acts as a central coordinator of the gene expression program that drives MCC differentiation, acting downstream of the Notch2-mediated lineage decision, and upstream of Multicilin. Phenotypic characterization of shTRRAP-treated primary human airway epithelial cells, together with RNA-seq and ChIP-seq data, indicate that TRRAP acts very early in MCC formation to control basal body generation and likely regulates multiple, additional steps of MCC differentiation and function by regulating a broad network of core MCC genes.

*TRRAP* was originally identified as an essential cofactor for the transformation activity of both c-Myc and E2F (McMahon et al., 1998). *TRRAP* is also important for stem/progenitor cell self-renewal and differentiation in multiple tissues. Conditional deletion of *Trrap* in mouse embryonic stem cells or the central nervous system led to a decrease in the expression of stemness genes or cell cycle regulators, respectively, and resulted in premature differentiation (Wurdak et al., 2010; Sawan et al., 2013; Tapias et al., 2014). Conditional deletion of *Trrap* in the hematopoietic system in adult mice led to p53-independent apoptosis and deregulation of *Myc* and resulted in a depletion of hematopoietic stem/progenitor cells (Loizou et al., 2009). Together, these studies indicate that *TRRAP* is required for maintaining stem/progenitor cells by keeping them in an undifferentiated state. Although we did not uncover a similar role for TRRAP in our study, we have observed TRRAP expression in proliferating basal cells in culture. Moreover, our shRNA reagents resulted in a partial TRRAP knockdown, which may have been insufficient to produce a pronounced phenotype in basal cells themselves. In contrast, we find that *TRRAP* is required for the differentiation of one of the major cell types in the airway, the MCC. These context-dependent functions of *TRRAP* may rely on the activity of tissue-specific transcription factors and need to be further explored in future studies. Interestingly, we did not find other components of the TRRAP-associated HAT complexes in our pooled shRNA screen. There are at least two possible reasons for this. First, the shRNA knockdown of other HAT complex components could be partial and insufficient to produce a phenotype. Second, TRRAP is a component of multiple distinct transcriptional coactivator complexes (Murr et al., 2007), and therefore knockdown of TRRAP could result in a stronger phenotype than knockdown of a factor that is specific to one coactivator complex.

We found that *TRRAP* acts downstream of Notch signaling and upstream of Multicilin to control MCC formation, rather than regulating the basal cell fate decision between secretory and ciliated cells. *TRRAP* knockdown had no effect on secretory cell production but blocked two key early events in MCC formation: basal body amplification and FOXJ1 expression. Our ChIP-seq and RNA-seq data indicate that TRRAP associates with the promoter for, and regulates the expression of, *MCIDAS*, the gene encoding for Multicilin, and the transcription factor *RFX3*. Multicilin has been shown to be a key transcriptional coregulator controlling MCC formation by regulating centriole assembly in both *Xenopus* embryonic epidermis and mouse tracheal epithelial cells (Stubbs et al., 2012), and Multicilin and Rfx3 are each required for Foxj1 expression during MCC formation in *Xenopus* epidermis and mouse ependymal cells, respectively (El Zein et al., 2009; Stubbs et al., 2012). We propose that TRRAP-mediated Multicilin and RFX3 expression are key events in the initiation of MCC differentiation.

Interestingly, neither TRRAP nor Multicilin acts as a transcription factor or has the ability to bind to DNA, but each has been reported to act together with E2F family members, in particular E2F4, to regulate gene expression. E2F4 is required for normal development of airway epithelium, as knockout mice have a significant reduction in ciliated cell numbers throughout the airway epithelium (Danielian et al., 2007). Moreover, *E2f4* mutation leads to chronic rhinitis and is associated with opportunistic bacterial infections in mice (Humbert et al., 2000). E2F4 forms a ternary complex with Dp1 and Multicilin (called the EDM complex) that regulates key genes for centriole biogenesis, and expression of a dominant-negative form of E2F4 blocks basal body formation in *Xenopus laevis* embryos (Ma et al., 2014). The transcriptional activation of *E2f4* is reported to require *TRRAP* (Lang et al., 2001). Overexpression of *TRRAP* in Cos1 cells significantly enhanced E2f4-dependent transcriptional activity. Moreover, overexpression of c-Myc, which competes with E2f4 for TRRAP’s binding site, repressed transcriptional activation by E2F4. Together with our findings, these data suggest that TRRAP may regulate E2F4-driven transcriptional activation by recruiting it, together with Multicilin and Dp, to specific loci labeled by histone modifications, or opening up the chromatin and allowing for the EDM complex to regulate expression. We are currently exploring the link between TRRAP, E2F, and Multicilin in regulating MCC formation.

Our shRNA screen also uncovered a role for *ATAD2B* in MCC differentiation. *ATAD2B* knockdown with individual shRNAs reduced the ratio of ciliated/secretory cells by 42% compared with shNT controls (Fig. 1 D). *ATAD2B* has an AAA ATPase domain and a bromodomain and is reported to play a role in neuronal differentiation and tumorigenesis (Leachman et al., 2010). In chick embryos, ATAD2B is transiently expressed in neuronal nuclei of the telencephalon and cerebellum. It has also been detected in multiple human tumors, including glioblastoma, oligodendroglioma, and breast carcinoma. Interestingly, *ATAD2*, a paralogue of *ATAD2B*, has been proposed as a coactivator for E2F proteins in cancer cell proliferation (Revenko et al., 2010). ATAD2 directly interacts with multiple E2F proteins, including E2F4, which is required for MCC formation (Danielian et al., 2007). E2F proteins are recruited to specific histone marks (H3K14ac) by binding to ATAD2B. Given the homology between *ATAD2* and *ATAD2B* (69% identical and 80% similar), it is possible that ATAD2B plays a role in recruiting of E2F proteins to certain genomic loci and activating MCC gene transcription.

Although the early block of MCC formation as a result of knocking down TRRAP can be explained by TRRAP regulation of MCIDAS and RFX3, our RNA-seq and ChIP-seq studies found that TRRAP directly regulates a broad network of genes involved in various aspects of MCC formation and function (Figs. 6 D and S3 D). It is worth noting that although more than 90% of the “core MCC genes” that are TRRAP DTGs are down-regulated after TRRAP knockdown, the expression of a small subset (9 of 112) is increased. The precise roles of each of these genes in MCC formation require further investigation. A particularly interesting group of TRRAP-regulated genes are those associated with PCD. We found that TRRAP regulates the expression of 19 of the 29 genes that have been described as being mutated in PCD (Horani and Ferkol, 2016). Although TRRAP is an essential gene (TRRAP knockout mice are not viable; Herceg et al., 2001), our knockdown data with shRNA suggest that a hypomorphic mutation could phenocopy mutations in one or more of the PCD genes and have profound effects on mucociliary clearance and chronic respiratory diseases.

## Materials and methods

### Pooled shRNA library design and construction

For detailed methods, see Hoffman et al. (2014). In brief, a 6,500-element shRNA library that includes 296 genes related to epigenetic regulation was cloned as a pool into the pRSI9 lentiviral plasmid (Cellecta). A mean of 17 uniquely barcoded shRNAs target each gene for measuring shRNA representation by NGS.

### Pooled shRNA screen

Normal human bronchial epithelial cells (HBECs; CC-2540; Lonza) were expanded twice with growth medium (500 ml BEGM medium [CC-3171; Lonza] and one SingleQuots kit [CC-4175; Lonza]) in T75 flasks to obtain passage 2 (P2) cells, which are >99% p63^+^ basal cells. ALI cultures using P2 cells were performed as previously described (Danahay et al., 2002). The packaged virus for the pooled shRNA library was added within 1 h after cell seeding at an MOI of 1. The cells were cultured in differentiation medium (250 ml BEGM medium, 250 ml DMEM [11965092; Thermo Fisher Scientific], and one SingleQuots kit lacking triiodothyronine, with a final concentration of 50 nM all-trans retinoic acid) on both apical and basal sides of Transwells for the first 7 d. Medium was removed from apical side, and cells were cultured for another 2 wk under ALI conditions. At day 21, cells were harvested with 0.05% Trypsin-EDTA (25300054; Thermo Fisher Scientific), fixed, and sorted based on FOXJ1 and ITGA6 signals following the modified MARIS protocol. The gDNA from sorted cells was extracted with RecoverALL Total Nuclei Acid Isolation kit (AM1975; Ambion) for NGS analysis.

### Data analysis for pooled shRNA screen

Library barcode counts from each sample were normalized to 12 million reads. The number of reads observed for each barcode in the ciliated population (FOXJ1^+^) was divided by the number of reads for the corresponding barcode in the secretory population (FOXJ1^−^, ITGA6^−^) to give the differential fold changes. A robust *z*-score was calculated using the median and mean absolute deviation across the log2 fold changes. To summarize the results at the gene level, we evaluated the statistical significance of all shRNAs, targeting each gene being unusually distributed toward the low end of the distribution (redundant siRNA activity [RSA] down) using the RSA algorithm (Konig et al., 2007). To visualize the gene significance and result strength, we plotted the RSA down value against the Q1 *z*-score for each gene to identify critical genes for ciliated cell formation.

### Lentiviral constructs

For gene knockdown, shRNA constructs targeting *TRRAP*, *ATAD2B*, *KDM6B*, and one nontargeting control oligo were generated using the oligos listed in Table S2. The oligos were annealed and then cloned into the pRSI16 vector (SVSHU616-l-CT; Cellecta) with BbsI digestion sites.

For overexpression, *MULTICILIN* (GeneID 345643) was cloned into the plenti6/V5-DEST.NGFP Gateway vector, which was generated by transferring the N-EmGFP ORF from pcDNA6.2/N-EmGFP-DEST (V35620; Thermo Fisher Scientific) into pLenti6/V5-DEST (V49610; Thermo Fisher Scientific).

### Lentivirus production

Lentiviral packaging of the pooled shRNA library was performed as previously described (Hoffman et al., 2014). For the viral packaging of individual shRNA constructs, 4 × 10^6^ 293T cells were seeded in a 100-mm poly-d-lysine–coated dish (Corning BioCoat; 356469) 1 d before transfection with 14 ml cell growth medium (DMEM, 10% FBS [631106; Clontech], 2 mM l-glutamine [25030; Invitrogen], 0.1 mM MEME nonessential amino acids [11140; Invitrogen], and 1 mM sodium pyruvate MEM [11360; Invitrogen]). For transfection, 7 µg packaging plasmid DNA (ViraPower lentiviral Packaging Mix, K497500; Thermo Fisher Scientific) was mixed with 5 µg expression construct DNA and 36 µl Fugene6 (E2691; Promega). OptiMEM (31985062; Thermo Fisher Scientific) was then added to the mixture for a total volume of 800 µl. 293T cells were incubated with the transfection reagent mixture for 24 h before the growth medium was refreshed. 72 h after transfection, virus was harvested and frozen for future experiments. For viral packaging of overexpression constructs (*GFP* and *GFP-MCIDAS*), the transfection procedure was performed as above, and then virus was concentrated with Lenti-X Concentrator (631231; Clontech) before freezing.

### Antibodies and reagents

Primary antibodies used in this study were mouse anti–acetylated α-tubulin (RRID: AB_609894; T7451; Sigma-Aldrich), mouse anti-MUC5AC (RRID: AB_2314822; MS-145-P0; Thermo Fisher Scientific), rabbit anti-MUC5B (RRID: AB_2282256; sc-20119, Santa Cruz Biotechnology), mouse anti-SCGB1A1 (MAA851Hu21; Cloud-Clone Corp.), rabbit anti-p63 (RRID:AB_2256361; 619001; BioLegend), rat anti–integrin α6 (RRID: AB_2128317; MAB1378; Millipore), mouse anti-pericentrin (RRID: AB_2160664; ab28144; Abcam), rabbit anti-TRRAP (RRID: AB_10672508; HPA038203; Sigma-Aldrich), rabbit anti-TRRAP (RRID: AB_10672042; ab73546; Abcam), and human anti-Notch2 (Danahay et al., 2015). Secondary antibodies (Alexa Fluor 488–, 568–, and 647–conjugated anti–mouse, anti–rabbit, and anti–rat antibodies; Thermo Fisher Scientific) were used in immunofluorescence (IF) and FACS. Prolong gold antifade with DAPI (P36935; Thermo Fisher Scientific) was used for nuclear staining.

### IF staining and imaging

ALI cultures of HBECs at different time points after seeding were washed with PBS twice and fixed with 4% PFA (15713; Electron Microscopy Sciences). Filters were washed with IF wash buffer (130 mM NaCl, 7 mM Na_2_HPO_4_, 3.5 mM NaH_2_PO_4_, 7.7 mM NaN_3_, 0.1% BSA, 0.2% Triton X-100, and 0.05% Tween-20) three times before blocking with block buffer (IF buffer with 10% goat serum) for 1 h. Cells were then incubated with primary antibodies overnight at 4°C, followed by 1-h secondary antibody incubation. For imaging, filters were punched out with 8-mm biopsy punches (33-37; Integra Miltex) and mounted in Prolong gold antifade containing DAPI for imaging. Images were collected on a confocal microscope imaging system that includes a microscope (Axiovert 200; Zeiss), a Yokogawa CSU-X1 spinning disc head, and an electron-multiplying charge-coupled device camera (Evolve 512; Photometrics). The images were captured with a 40× objective (Plan-Apochromat 40×/1.3 Ph3 M27; Zeiss) and collected with Zen blue software (Zeiss).

Cell Profiler (Kamentsky et al., 2011) was used for image analysis. The analysis pipeline was first optimized to set the parameters for measuring cells of interest on a subset of images and then applied to the entire dataset with all of the parameters maintained constant.

For the data shown in Figs. 2 F and 4 D, the procedure was as follows. (1) Read in images and split into two channels: transfected (RFP-expressing cells) and individual cell type–specific markers (antibody stained). (2) Identified RFP^+^ transfected cells with IdentifyPrimaryObjects module in the RFP channel. Globally thresholded image using Otsu two classes, correction factor 1.2, and object size 20–200-pixel diameter. (3) Used ApplyThreshold module to convert cell type–specific marker image to binary (black and white). Choose threshold strategy as automatic. (4) Identified cell type–specific marker expressing cells with IdentifyPrimaryObjects module from the converted signals from step 3. Thresholding was set to manual, and object size was set between 20–100 or 20–200 pixels in diameter, depending on the cell type–specific marker measured. (5) Identified RFP^+^ cells that express the cell type–specific marker with RelateObjects module. Set the input child objects as cell type–specific marker objects identified in step 4 and the input parent objects as the RFP objects identified in step 2. (6) Further improve the measurement by using FilterObjects module. Set the filtering mode as measurements, and select the filtering method with limits. The percentage of RFP^+^ cells expressing the cell type–specific marker was calculated by dividing the number of objects identified in step 6 by the number of total RFP^+^ cells identified in step 2.

For the data shown in Fig. S2 D, the procedure was as follows. (1) Read in images and split into three channels, nuclear (DAPI staining), transfected (RFP-expressing cells), and TRRAP (antibody stained). (2) Globally thresholded image based on TRRAP signal using Otsu three-class thresholding, where the middle class was set to the background and the threshold correction factor was 1.2. (3) Identified RFP^+^ (transfected) cells with IdentifyPrimaryObjects module in the RFP channel. Thresholding was set to robust background, correction factor to 1.2, and object size to 15–50-pixel diameter. (4) Identified nuclei of RFP^+^ cells with IdentifySecondaryObjects module in the DAPI channel. Thresholding was set to Otsu two-class thresholding, correction factor 0.8. (5) Created masked images of RFP^+^ (transfected) and RFP^−^ (nontransfected) cells using the objects found in step 4. (6) Identified all nuclei in either RFP^+^ or RFP^−^ cells with IdentifyPrimaryObjects module in the TRRAP channel on the masked images. Thresholding was set to OTSU three-class thresholding with the middle class set to background, correction factor 1.1. The object size was set to 15–60-pixel diameter. (7) Identified TRRAP^+^ nuclei in either RFP^+^ or RFP^−^ cells with IdentifyPrimaryObjects module in the TRRAP channel on the masked images. Thresholding was set to Otsu three-class thresholding with middle class set to background, correction factor 1. The object size was set to 15–60-pixel diameter. From these found objects, the percentage of TRRAP-positive cells was calculated in either the RFP^+^ or RFP^−^ population.

### Flow cytometry and cell sorting

Cells were harvested by Trypsin and washed with PBS once before fixation with 4% PFA. After blocking with block buffer (IF buffer with 1% BSA) for 30 min, cells were stained with FOXJ1 (RRID: AB_1078902; HPA005714; Sigma-Aldrich) and ITGA6 (RRID: AB_2128317; MAB1378; Millipore) antibodies for 30 min, followed by incubation with secondary antibodies (Alexa Fluor 488 anti-rat and 647 anti-rabbit). The cells were suspended in 2% FBS and analyzed by flow cytometry or cell sorting. When RNA was required to be extracted and recovered after cell sorting, RNase inhibitors (N2611; Promega) were included in all of the above buffers at a concentration of at least 1:100.

### RNA isolation and qPCR

RNA from fixed cells was extracted with RecoverALL Total Nuclei Acid Isolation kit (AM1975; Thermo Fisher Scientific). RNA from the nonfixed samples was extracted with Trizol (15596026; Invitrogen). cDNA was synthesized from 1 µg RNA with qScript XLT cDNA Super Mix kit (95161-100; Quanta Biosciences). qPCR was performed using FastStart Universal Probe Master kit (04914058001; Roche) with 40 ng cDNA per reaction. Taqman probes for qPCR (Applied Biosystems) are as follows: *FOXA3*, Hs00270130_m1; *FOXJ1*, Hs00230964_m1; *P63*, Hs00978340_m1; *GAPDH*, Hs99999905_m1; and *TRRAP*, Hs00268883_m1.

### ChIP

Day 21 ALI cultures from two independent donors were fixed with 1% formaldehyde for 15 min and quenched with 0.125 M glycine. Samples were sent to Active Motif for ChIP-seq and ChIP-qPCR. Chromatin was isolated by the addition of lysis buffer, followed by disruption with a Dounce homogenizer. Lysates were sonicated, and DNA was sheared to a mean length of 300–500 bp. Genomic DNA (input) was prepared by treating aliquots of chromatin with RNase, proteinase K, and heat for de-cross-linking, followed by ethanol precipitation. Pellets were resuspended, and the resulting DNA was quantified on a NanoDrop spectrophotometer. Extrapolation to the original chromatin volume allowed quantitation of the total chromatin yield.

An aliquot of chromatin (30 µg) was precleared with protein A agarose beads (Invitrogen). Genomic DNA regions of interest were isolated using 6 µg antibody against TRRAP (RRID: AB_10672042; ab73546; Abcam). Complexes were washed, eluted from the beads with SDS buffer, and subjected to RNase and proteinase K treatment. Cross-links were reversed by incubation overnight at 65°C, and ChIP DNA was purified by phenol-chloroform extraction and ethanol precipitation.

### ChIP sequencing (Illumina) and ChIP-qPCR

Illumina sequencing libraries were prepared from the ChIP and input DNAs by the standard consecutive enzymatic steps of end-polishing, dA addition, and adaptor ligation. After a final PCR amplification step, the resulting DNA libraries were quantified and sequenced on Illumina’s NextSeq 500 (75-nt reads, single end). The raw data are available in the National Center for Biotechnology Information Sequence Read Archive under accession no. SRP107101.

Analysis was performed with the AQUAS TF ChIP-seq pipeline (https://github.com/kundajelab/chipseq_pipeline) using type TF and species hg19; all other parameters remained as the default. In brief, the workflow aligns reads to the human reference genome (hg19) with BWA (Li and Durbin, 2009; v.0.7.4), and duplicate reads are marked with Picard “mark duplicates” (v.2.8.1; https://broadinstitute.github.io/picard/) and removed with SAMtools (Li et al., 2009; v.1.3.1). Peaks locations were determined using MACS2 (Zhang et al., 2008; v.2.1.0.20150603) and SPP (Kharchenko et al., 2008) algorithms. Peaks that were reproducible between and within replicates were identified using the irreproducible discovery rate (IDR; Li et al., 2011; https://github.com/nboley/idr). The optimal peaks, or those with a global IDR <0.05 that did not overlap with black-listed peaks, were used in downstream analyses.

Genomic signal maps of the peak fold change and p-values were derived and stored in bigwig files for visualization. The fold change was calculated using the MACS2 algorithm and is the signal enrichment for a region divided by the local background.

Genomic annotations (GRCh37) were assigned to each peak using ChIPseeker (Yu et al., 2015; v.1.8.9). Genes were defined as being TRRAP occupied if the genomic location of a peak overlapped the promoter of a gene (transcription start site ± 1,000 bp). Detected TRRAP peaks, with genomic annotation, are listed in Table S5.

qPCR reactions were performed in triplicate using SYBR Green Supermix (170-8882; Bio-Rad) on a CFX Connect Real Time PCR system. One positive control site (DDX5), one negative control that amplifies a region in a gene desert on chromosome 12 (Untr12; 71001; Active Motif), and four test sites of interest were tested. Untr12 was identified as a region on the genome that has no known gene annotations within 100–200 kb and is thus not expected to bind any transcription factors. The resulting signals were normalized for primer efficiency by carrying out qPCR for each primer pair using input DNA (pooled unprecipitated gDNA from each donor). Fold enrichment was calculated as [normalized value (test region A)/normalized value (Untr12)]_TRRAP ChIP_/[normalized value (test region A)/normalized value (Untr12)]_IgG ChIP_. The following test site primer sets were used: MCIDAS: forward, 5′-GAAGCCAGCCAGAGGTTG-3′, and reverse, 5′-CCATCTCTCAGCACCTCCTC-3′; CCNO: forward, 5′-CGGGTGGCCGCTTTACTAC-3′, and reverse, 5′-ACTTTCGAGTGCGCGTTTG-3′; MYB: forward, 5′-TGCTGGATGCATTGAGATATG-3′, and reverse, 5′-CCAGCATTTTCCCTGTATCTG-3′; and RFX3: forward, 5′-AGCTATGGCGAGGGAAGAATC-3′, and reverse, 5′-GCCCCGGAAGTAACGTATC-3′.

### RNA sequencing

Gene expression for shNT-, shTRRAP-a–, and shTRRAP-b–expressing cells at ALI days 4 and 14 was measured using RNA sequencing technology. Each experimental condition was performed in duplicate with two independent donors. Cells expressing the shRNA constructs (RFP^+^) were sorted by FACS, and RNA was isolated with Trizol and cleaned up with the RNeasy kit (74106; Qiagen). The amount of RNA was quantified with an RNA 6000 Nano kit (5067-1511; Agilent Technologies).

RNA libraries were prepared using the Illumina TruSeq Stranded mRNA Sample Preparation kit and sequenced using the Illumina HiSeq2500 platform following the manufacturer’s protocol. Samples were sequenced in paired-end mode to a length of 2× 76 bp. Images from the instrument were processed using the manufacturer’s software to generate FASTQ sequence files. Read quality was assessed by running FastQC on the FASTQ files. Sequencing reads showed excellent quality, with a mean Phred score higher than 30 for all base positions. A mean of 49 million 76-bp read pairs were mapped to the *Homo sapiens* genome (GRCh37) and the human gene transcripts from Ensembl v75 (Cunningham et al., 2015) by using an in-house gene quantification pipeline (Schuierer and Roma, 2016). A mean of more than 98% of the total reads were mapped to the genome or the transcripts, and more than 90% to the exons and junctions (expressed reads). Genome and transcript alignments were used to calculate gene counts based on Ensembl gene IDs. The raw RNA-sequencing reads are available in the National Center for Biotechnology Information Sequence Read Archive under accession no. SRP106050.

Additional quality control was performed using Picard, which indicated that all samples were of consistently high quality. Genes with mean counts per million less than 1 for all of the shRNA treatment/time-point groups were excluded from further analysis. Differential expression analysis was performed on the counts using voom normalization followed by limma (Ritchie et al., 2015) for model-fitting with R v.3.1.3. PCA analysis on voom-normalized counts indicated that PC1 contained 23% of the variance and separated shTRRAP from nontargeted shRNA; PC2 contained 21% of the variance and reflected each TRRAP shRNA (i.e., shTRRAPa vs. shTRRAPb); and PC3 contained 16% of the variance and reflected time point. Because donor differences were not reflected in the first three principle components and contributed to a relatively small amount of variance compared with shRNA treatment and time, the linear model consisted of only two factors: shRNA (levels: shTRRAP-a, shTRRAP-b, shNT) and time point (levels: 4 or 14 d). Differential expression was performed using a contrast matrix with limma, and results for all genes and all comparisons are included in Table S4. TRRAP DEGs were defined as those that met the following criteria. At either time point, a fold change of at least 1.5 (in either direction) and Benjamini–Hochberg adjusted p-value <0.05 with either shTRRAP-a or shTRRAP-b. If differential expression was observed with only one of the shRNAs, we required that the gene be responsive to the other TRRAP shRNA with a fold change of at least 1.2 in the same direction. DEGs were restricted to protein coding, as denoted by gene biotype in Ensembl v.75, and are listed in Table S3.

### Gene set comparison

Gene lists contained sets of MCC genes (supplemental tables 1 and 2 in Hoh et al., 2012; supplemental table 2, tab 3 (“Cilia-related genes mouse”), in Nemajerova et al., 2016; SysCilia (http://www.syscilia.org/goldstandard.shtml); supplemental table 7, tab 4, in Quigley and Kintner, 2017), PCD genes (table 1 in Horani and Ferkol, 2016), and airway basal cell genes (supplemental table 2 in Hackett et al., 2011). The gene lists used are listed in Table S6. For nonhuman lists, orthologues were identified using mappings from the Jackson Laboratory (Mouse Genome Informatics). Gene lists were restricted to protein coding genes and Ensembl v.75 annotations. Enrichment for the sets, compared with DEGs and TRRAP-occupied genes, was calculated using Fisher’s exact test for count data (R v.3.3.0, fisher.test). P-values were adjusted for multiple testing using the Bonferroni correction (R v.3.3.0, p.adjust; p-value, method = bonferroni, *n* = 6). The gene universe used for all enrichments was 22,810, the number of protein coding genes in Ensembl v.75.

### Online supplemental material

Fig. S1 describes the validation of the sorting method used for the pooled shRNA screen and shows the individual channels for the images in Fig. 2 E. Fig. S2 shows the validation of the TRRAP antibody used for IF studies in Fig. 3. Fig. S3 shows the individual channels for the images in Fig. 4 C, the ChIP-qPCR data of TRRAP binding to the regulatory regions of four genes identified as TRRAP DTGs shown in Fig. 6 E, and a model of how TRRAP regulates multiciliated formation. Table S1 contains a list of genes targeted by the epigenetics pooled shRNA library. Table S2 contains a list of sequences used for the shRNA constructs used in this study. Table S3 contains a list of genes that are differentially expressed after TRRAP knockdown. Table S4 contains the RNA-seq expression data used to generate Fig. 6. Table S5 contains the ChIP-seq data used to generate Fig. 6. Table S6 contains the published cilia and basal cell gene lists used for set enrichment analysis in Fig. 6.

## Supplementary Material

Supplemental Materials (PDF)

Tables S1-S6 (ZIP)
