# Supplementary material for: TRRAP is a central regulator of human multiciliated cell formation
Source: J Cell Biol. 2018 Jun 4;217(6):1941–55. doi: 10.1083/jcb.201706106 (PMC5987713; doi:10.1083/jcb.201706106)
Supplement: Supplemental Materials (PDF) [file JCB_201706106_sm.pdf]

Supplemental material

Wang et al., https://doi.org/10.1083/jcb.201706106

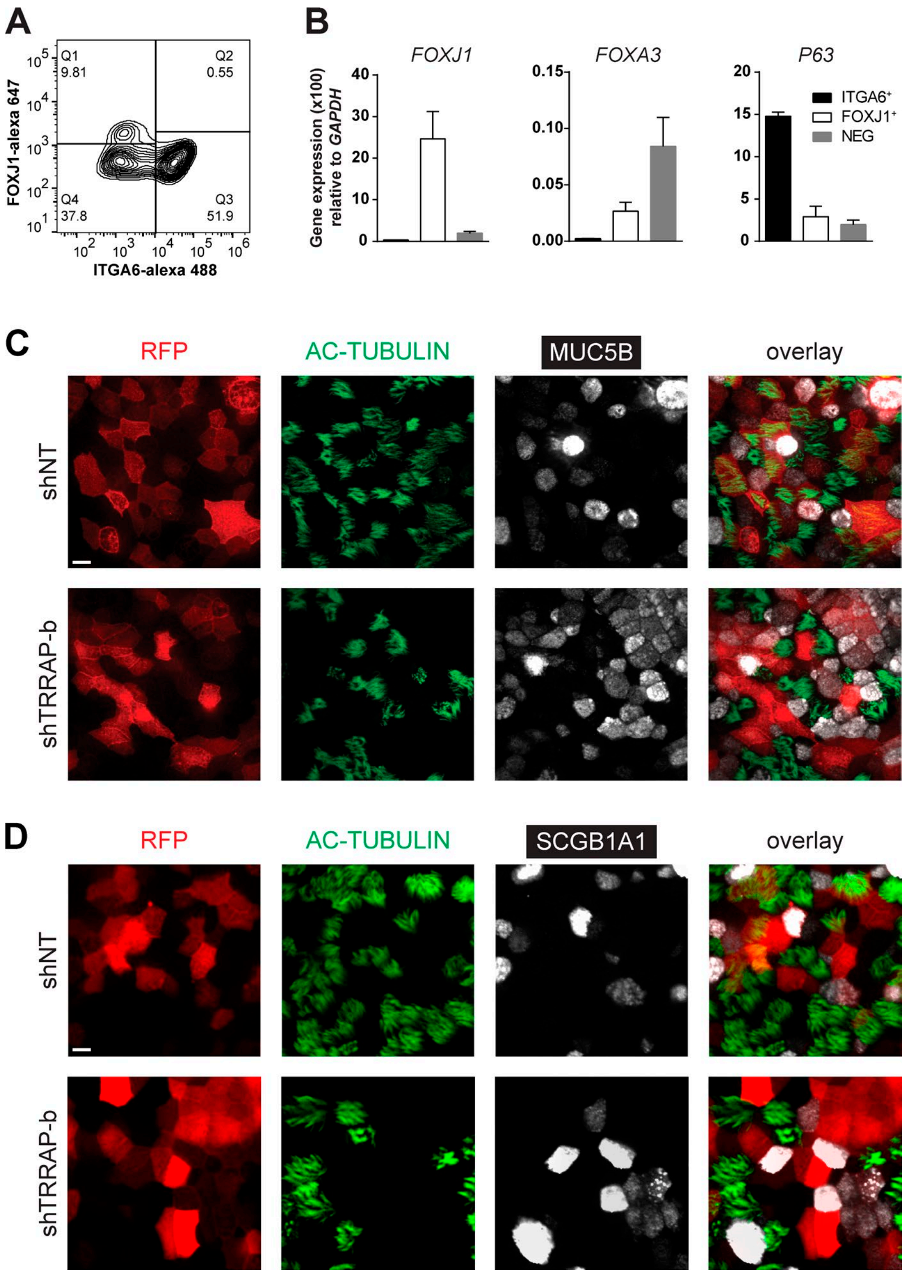

Figure S1. **A pooled shRNA screen identifies a requirement for TRRAP in MCC formation. (A and B)** Validation of the sorting method for the pooled shRNA screen. **(A)** A representative FACS plot from day 21 (ALI D14) cultures stained with FOXJ1 and ITGA6 antibodies. **(B)** Quantitative PCR analysis of ciliated (*FOXJ1*), goblet (*FOXA3*), and basal cell markers (*P63*) from day 21 (ALI D14) cultures sorted for FOXJ1 (ciliated; $FOXJ1^+$ $ITGA6^-$) or P63 (basal; $FOXJ1^-$ $ITGA6^+$) markers. n = 3; mean ± SEM. **(C and D)** TRRAP is required for MCC formation. Representative images of ALI cultures transduced with either shNT or shTRRAP-b (RFP^+). Cultures were stained with antibodies to acetylated α-tubulin (AC-TUBULIN) to label ciliated cells and MUC5B (A) or SCGB1A1 (B) to label goblet or club cells, respectively. Bars, 10 µm.

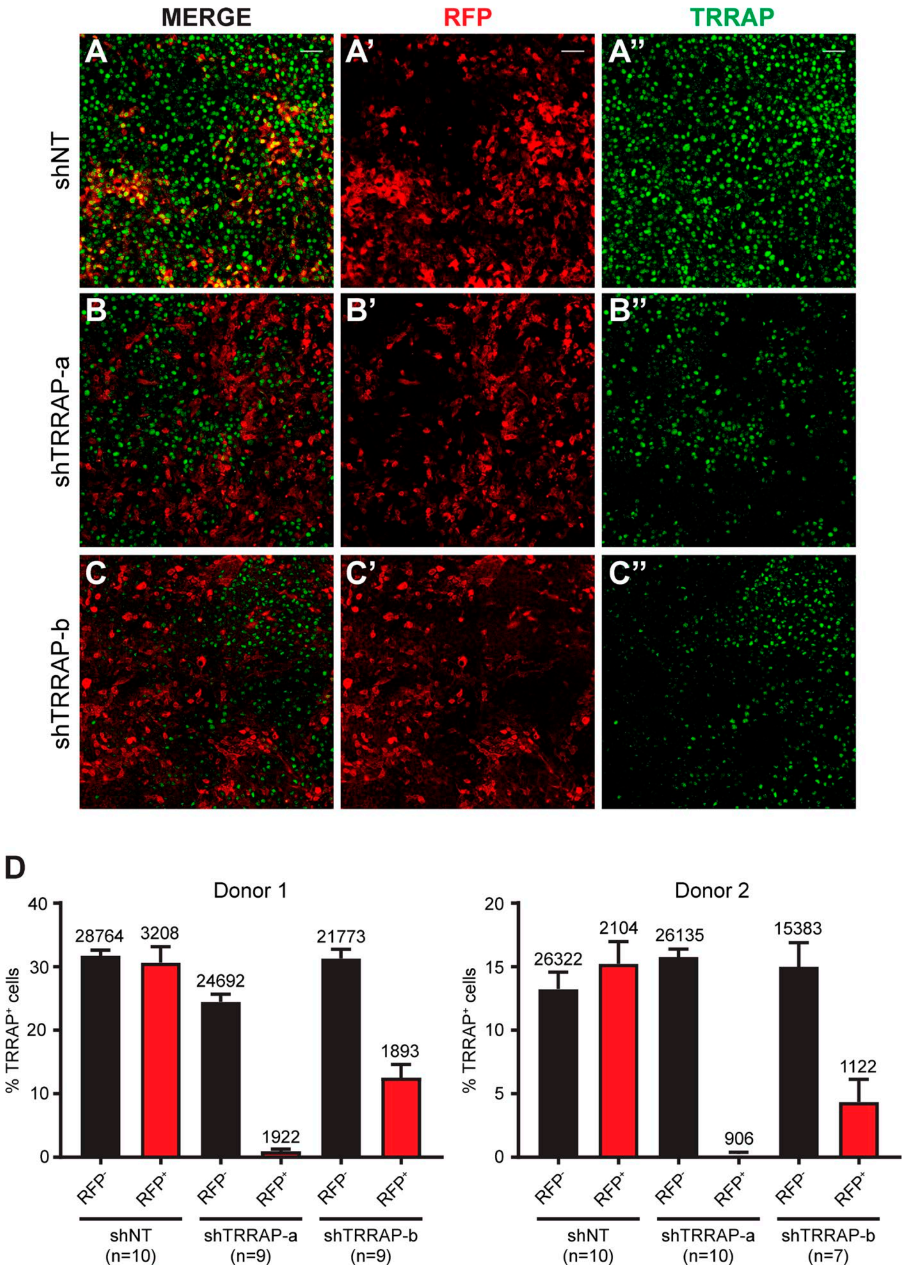

Figure S2. **TRRAP antibody validation. (A–C)** Representative images of ALI cultures transduced with shNT, shTRRAP-a, or shTRRAP-b. Cultures were fixed and stained with anti-TRRAP antibody at day 21 (ALI D14). Bars, 50 µm. **(D)** Quantification of the percentage of $TRRAP^+$ cells in cultures from two independent donors transduced with shNT, shTRRAP-a, or shTRRAP-b. Shown is the percentage of $TRRAP^+$ cells within the RFP^- (untransduced) and RFP^+ (transduced) populations in for each treatment. Data are presented as mean ± SEM. The number of fields (*n*) is shown below, and the total number of cells analyzed for each condition is shown above.

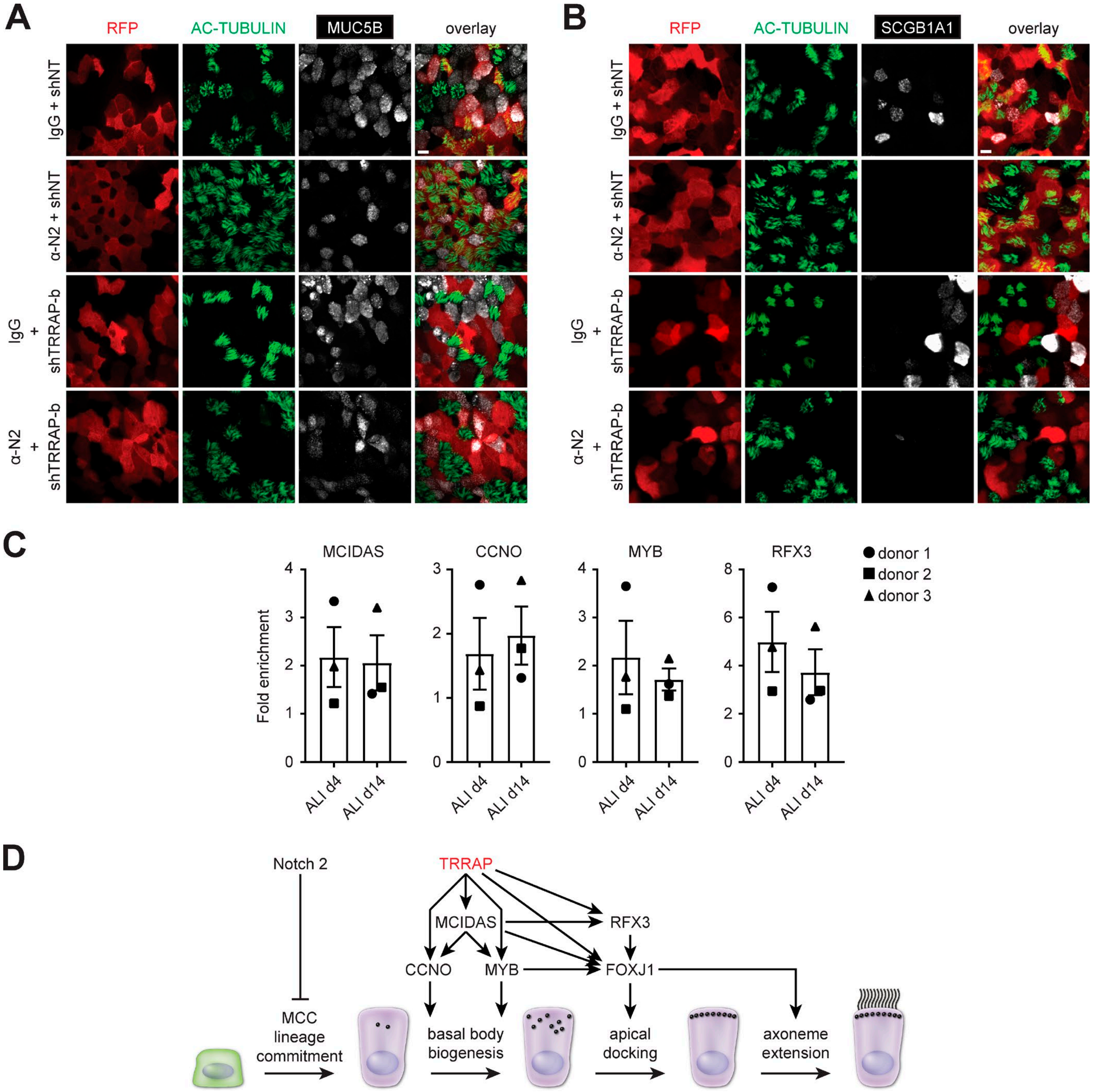

Figure S3. **TRRAP acts downstream of Notch2 in MCC formation and binds to the regulatory regions of core ciliogenesis genes. (A and B)** Representative images of ALI cultures transduced with shNT or shTRRAP-b. Human airway basal cells were infected with shRNA viruses at day 0, then cultured at ALI in media containing either 1 µg/ml IgG or α-N2 from day 7. 2 wk after antibody treatment, cells were fixed and stained with AC-TUBULIN to label ciliated cells and MUC5B (A) or SCGB1A1 (B) to label goblet or club cells, respectively. Bar, 10 µm. **(C)** ChIP-qPCR of the regulatory regions of the indicated genes at ALI days 4 and 14. Data are presented as fold enrichment versus an untranscribed genomic region and IgG controls for three independent donors ($n = 3$; mean ± SEM). **(D)** Model of TRRAP regulation of MCC formation. TRRAP acts downstream of the MCC lineage decision, upstream of a transcriptional cascade controlling multiple steps of multiciliogenesis. The phenotypic consequence of TRRAP knockdown reflects the earliest requirement of TRRAP activity in MCC formation: centriole multiplication.

Provided online are six tables in Excel. Table S1 is a list of genes targeted by the epigenetics pooled shRNA library. Table S2 shows sequences used for shRNA constructs. Table S3 is a list of DEGs. Table S4 shows RNA-seq expression data and differential expression. Table S5 shows ChIP-seq data. Table S6 is the cilia- or basal-cell gene lists.
